# Age-Related Changes in Corneal Deformation Dynamics Utilizing Scheimpflug Imaging

**DOI:** 10.1371/journal.pone.0140093

**Published:** 2015-10-13

**Authors:** Marta E. Rogowska, D. Robert Iskander

**Affiliations:** Department of Biomedical Engineering, Wroclaw University of Technology, Wroclaw, Poland; Casey Eye Institute, UNITED STATES

## Abstract

**Purpose:**

To study age-related changes in corneal deformation response to air-puff applanation tonometry.

**Methods:**

Fifty healthy subjects were recruited for a prospective study and divided into two equal age groups (≤ 28 and ≥ 50 years old). Up to three measurements by a corneal deformation analyser based on the Scheimpflug principle were performed on the left eye of each subject. Raw Scheimpflug images were used to extract changes in anterior and posterior corneal profiles, which were further modelled by an orthogonal series of Chebyshev polynomial functions. Time series of the polynomial coefficients of even order exhibited a dynamic behavior in which three distinct stages were recognized. A bilinear function was used to model the first and the third stage of corneal dynamics. Slope parameters of the bilinear fit were then tested between the two age groups using Wilcoxon rank sum test and two-way non-parametric ANOVA (Friedman) test.

**Results:**

Statistically significant changes (Wilcoxon test, *P*<0.05) between the age groups were observed in the phase of the second applanation dynamics for the posterior corneal profile. In a two-way comparison, in which the corneal profile was used as a dependent variable, statistically significant changes (ANOVA/Friedman test, *P* = 0.017) between the groups were also observed for that phase.

**Conclusion:**

Corneal biomechanics depend on age. The changes in corneal deformation dynamics, which correspond to mostly free return of the cornea to its original shape after the air pulse, indicate that the age related differences in corneal biomechanics are subtle but observable with high speed imaging.

## Introduction

The composition and function of the human eye have been studied for decades. However, there is still much to be discovered in relation to the structure and properties of the individual components of the eye and changes in these properties as a result of aging processes. One of the most important components of the human eye is the cornea with about 70 per cent of the optical power [1, 2]. The structure of the cornea is defined as composite and it is currently considered to consist of six layers: epithelium, Bowman’s layer, stroma, Dua’s layer, Descemet’s membrane, and endothelium [3]. The stroma fibrous form almost 90 per cent of the total corneal thickness and determine the biomechanical behaviour of the cornea [4, 5]. It is well known that the cornea has viscoelastic material properties, which means that it has both elastic and viscous properties [6, 7]. The loading and unloading processes of viscoelastic materials by external force are not time reversible and are characterized by the occurrence of hysteresis. It is also known that age affects material properties of the cornea [4, 8–14]. A recent study of Elsheikh et al. [4] has shown strong statistical association between corneal stiffness and age. They proposed a model relating the two factors that is suitable for implementation in numerical simulations of ocular biomechanical behavior.

Assessing corneal biomechanical properties is important for a number of applications including tonometry measurement [15–18], which is used in ophthalmological management of glaucoma, corneal refractive surgeries [19, 20], injury treatment [21] and contact lens wear [22].

Recently, an air puff system using ultra-high speed Scheimpflug camera has been proposed as a potential tool to determine and register corneal biomechanical properties [23, 24]. The Scheimpflug analyser (Oculus, Wetzler, Germany) utilizes a rapid and symmetrically metered air pulse to deform the cornea (pressure range: 1 mmHg to 60 mmHg) [25]. The built-in ultra-high speed Scheimpflug camera is used to image the corneal deformation response at 4,330 frames per second covering a horizontal distance of about 8 mm. A total of 140 images of horizontal cross-section of the cornea are acquired (140/4330 ≈ 32 ms). Each image has 576 measuring points in the horizontal direction. Scheimpflug camera module of Scheimpflug analyser has blue light LED (455 nm, UV free) and records the corneal deformation dynamics in response to non-contact air-puff tonometry [24, 25].

**Fig 1:**
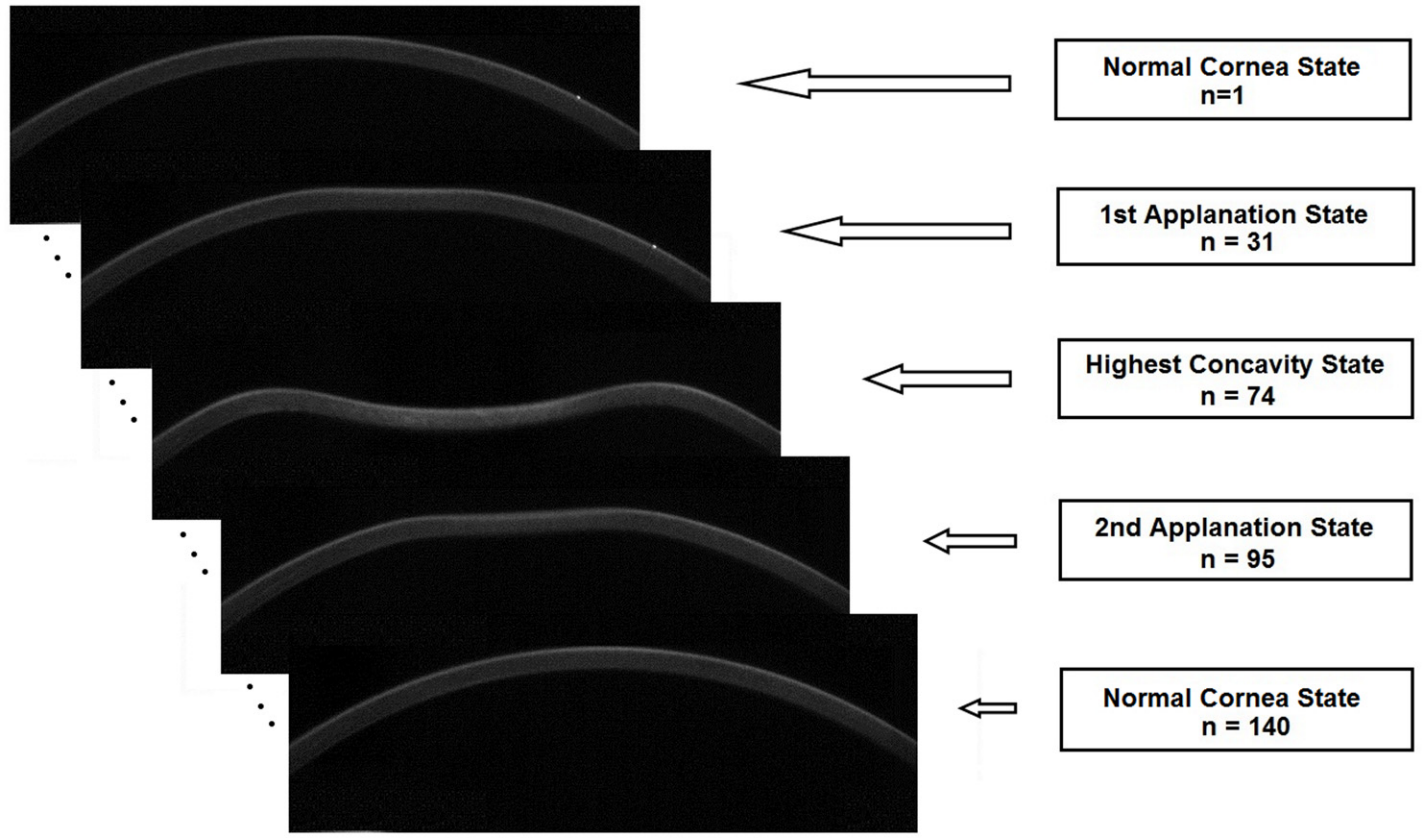
Changes in corneal shape for a few sample frames. The first frame refers to the normal cornea state, the next frames are associated with the first applanation state (n = 31), the concavity state (n = 74), and the second applanation state (n = 95) while the last frame corresponds to return of the cornea to its initial state.

The Scheimpflug images present the dynamic aspect of anterior and posterior corneal surface deformation. Fig 1 presents the changes in the corneal profile for the few sample frames. The first frame (n = 1) refers to the normal cornea state, next frames are associated with the first applanation state (n = 31), the highest concavity state (n = 74), and the second applanation state (n = 95) while the last frame (n = 140) corresponds to return of the cornea to its initial state.

Having a detailed picture of corneal deformation during measurement, the output of Scheimpflug analyser can be utilized to assess biomechanical characteristics of the cornea. The goal of this study was to explore the corneal deformation dynamics recorded in raw Scheimpflug images to evaluate age-related changes in corneal biomechanics in subjects with healthy corneas.

## Methodology

### Subjects and measurements

This was a prospective study in which two groups of subjects (28 males and 22 females) were considered, including young group of 25 subjects, aged from 20 to 28 (23 ± 3, mean ± SD) years and an older group of 25 subjects, aged from 50 to 66 (58 ± 5, mean ± SD) years. Each participant underwent examination, including review of medical history, slit lamp biomicroscopy, and finally, corneal deformation response measurements with Scheimpflug analyser. Exclusion criteria included history of any corneal pathology, signs of dry eye, eye surgery, any systemic disease and contact lens wear. For each subject, up to three measurements of corneal deformation response (in terms of high speed Scheimpflug camera recordings) were carried out on left eyes only. Each acquired series of images was saved for further analysis. To assess uniformity of subjects in age groups, central corneal thickness (CCT) and the intraocular pressure (IOP) registered by Scheimpflug analyser was noted.

All measurements were performed in succession, allowing about one minute break between each acquisition. To take into account diurnal variations in subjects IOP [26] all measurements were conducted in the mornings between 10 am and 12 pm.

The study adhered to the tenets of the Declaration of Helsinki and was approved by the Ethics Committee of the Medical University of Wroclaw (KB 503/2011 agreement). Written informed consent was obtained from all participants included in this study. Patient records were anonymized and de-identified prior to analysis.

### Data analyses and statistical methods

**Fig 2:**
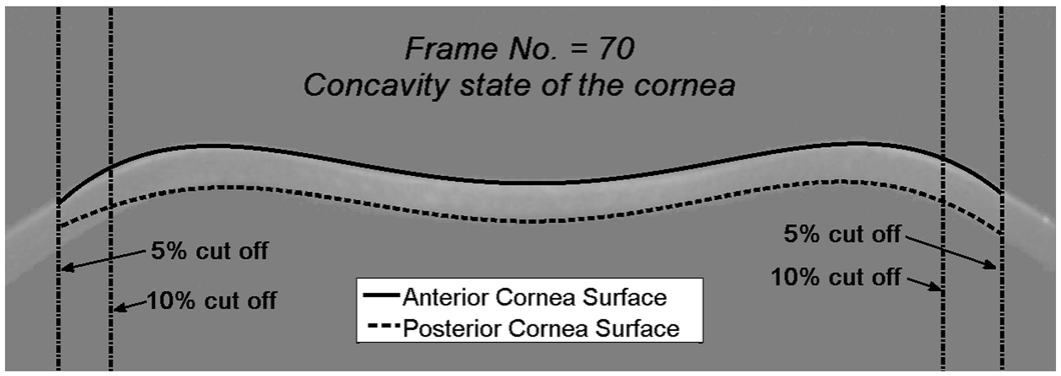
An example frame (n = 70) for the concavity state of the cornea with Chebyshev polynomial approximation of anterior and posterior cornea profiles.

The raw Scheimpflug images were numerically processed using a custom-written program in Matlab (Math Works, Inc., Natick, MA, USA). Segmentation algorithms including Otsu’s thresholding method [27] for edge detection of the anterior and posterior corneal contours were applied for every frame. Because of increasing noise at the cornea periphery, two values of data trimming were considered. This corresponded to cutting out either 5% or 10% of the extracted profile data on both sides. Final analysis was performed for a 10% cut that showed to be more robust in terms of the subsequent parametric modelling of the corneal edge data. Fig 2 presents a sample frame for concavity state of the cornea with polynomial approximation of anterior (solid line) and posterior (dashed line) cornea surfaces.

In the preliminary analysis on a smaller group of subjects [28], standard polynomial approximation with the optimally set order of six (in the Akaike Information Criterion sense [29]) was performed for detected edges. The results of this analysis were not entirely conclusive when the extended set of data was used. Subsequently, this was followed with Chebyshev polynomial approach as they form a set of orthogonal and complete series of basis functions in which estimated coefficient is independent of the other in the series [30]. More importantly, the boundary behavior of Chebyshev polynomials at the edges of the extracted edges is more stable. Time varying polynomial Chebyshev coefficients *a*_0_(*t*),*a*_1_(*t*),…,*a*_*n*_(*t*), were estimated for a given time instant *t* using a least squares procedure from a model:

$$p_{c}\left(x,t\right)=\;a_{n}\left(t\right)T_{n}\left(x\right)+a_{n-1}\left(t\right)T_{n-1}\left(x\right)+\ldots +a_{1}\left(t\right)T_{1}\left(x\right)+a_{0}\left(t\right)T_{0}\left(x\right)+\varepsilon \left(t\right),$$

**Fig 3:**
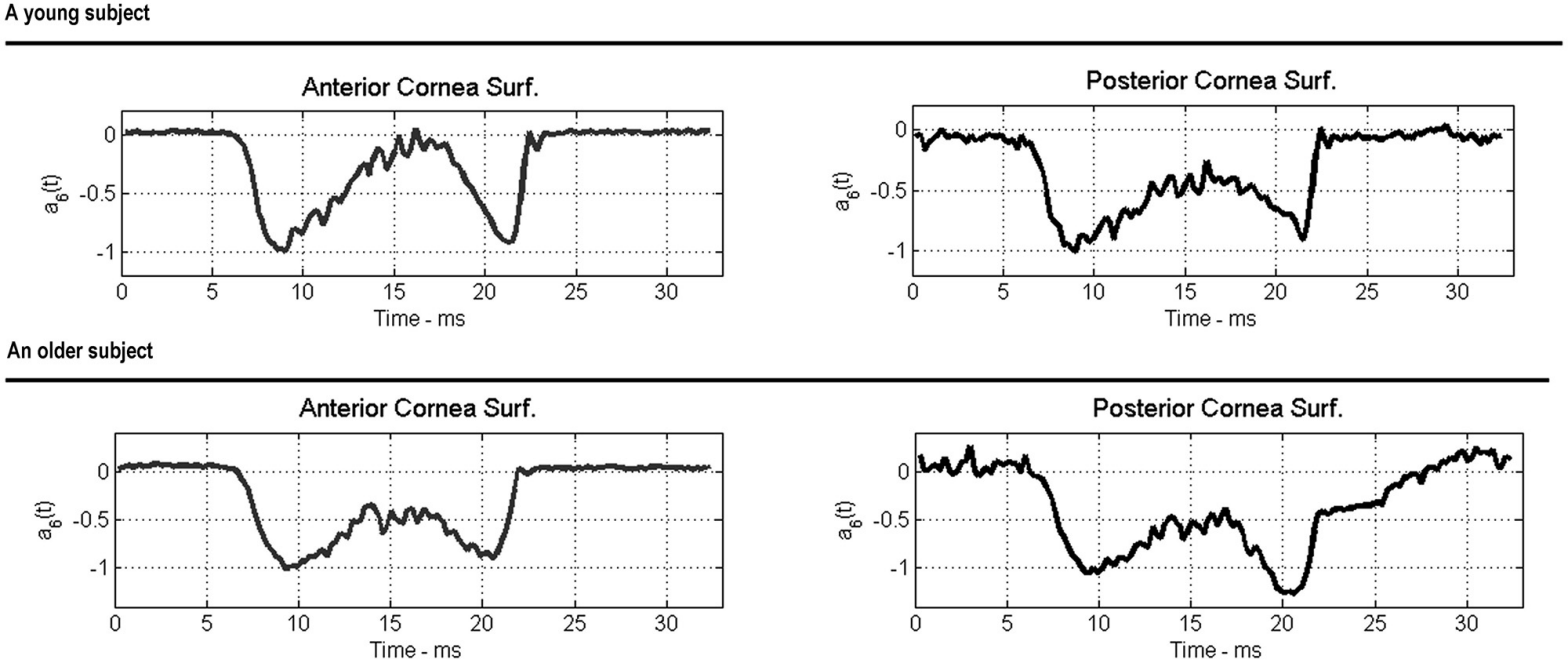
Examples of the time-varying Chebyshev polynomial coefficient *a*_6_(*t*) for selected individuals from the young (top) and older (down) group of subjects.

Where *T*_*k*_(*x*), *k* = 0,1,…,*n*, calculated recursively,

$$\begin{array}{c} T_{0}\left(x\right)=1\\ T_{1}\left(x\right)=x\\ \vdots \\ T_{n+1}\left(x\right)=2xT_{n}\left(x\right)-T_{n-1}\left(x\right) \end{array}$$

is the *k*-th Chebyshev polynomial with *x* representing the horizontal image axis in pixels, and *ε*(*t*) denotes the measurement and modeling error. Similarly as in the case of standard polynomials, Akaike Information Criterion was used to determine the optimal model order, whose median for all considered measurements was equal to six. Fig 3 shows an example of the time-varying Chebyshev polynomial coefficients, *a*_6_(*t*), describing changes in anterior and posterior corneal surfaces for two selected subjects from the young and older group. Similar time variation of corneal deformation was observed for all even order Chebyshev polynomial coefficients, i.e., for *a*_2_(*t*) and *a*_4_(*t*).

For each modelled measurement consisting of about 32 ms time series of the sixth order Chebyshev polynomial coefficient a three-stage process was observed. The first stage comprises the time from the first recorded image to the maximum concavity state (from about 7 to 11 ms from the start of the recording). The second phase describes the corneal oscillation period [24] (from about 11 to 16 ms) while the third stage corresponds to outgoing concavity and return of the cornea to its initial state (corresponds to the rest of the 32 ms recording). In this study, the first and the third stage of that process is considered.

**Fig 4:**
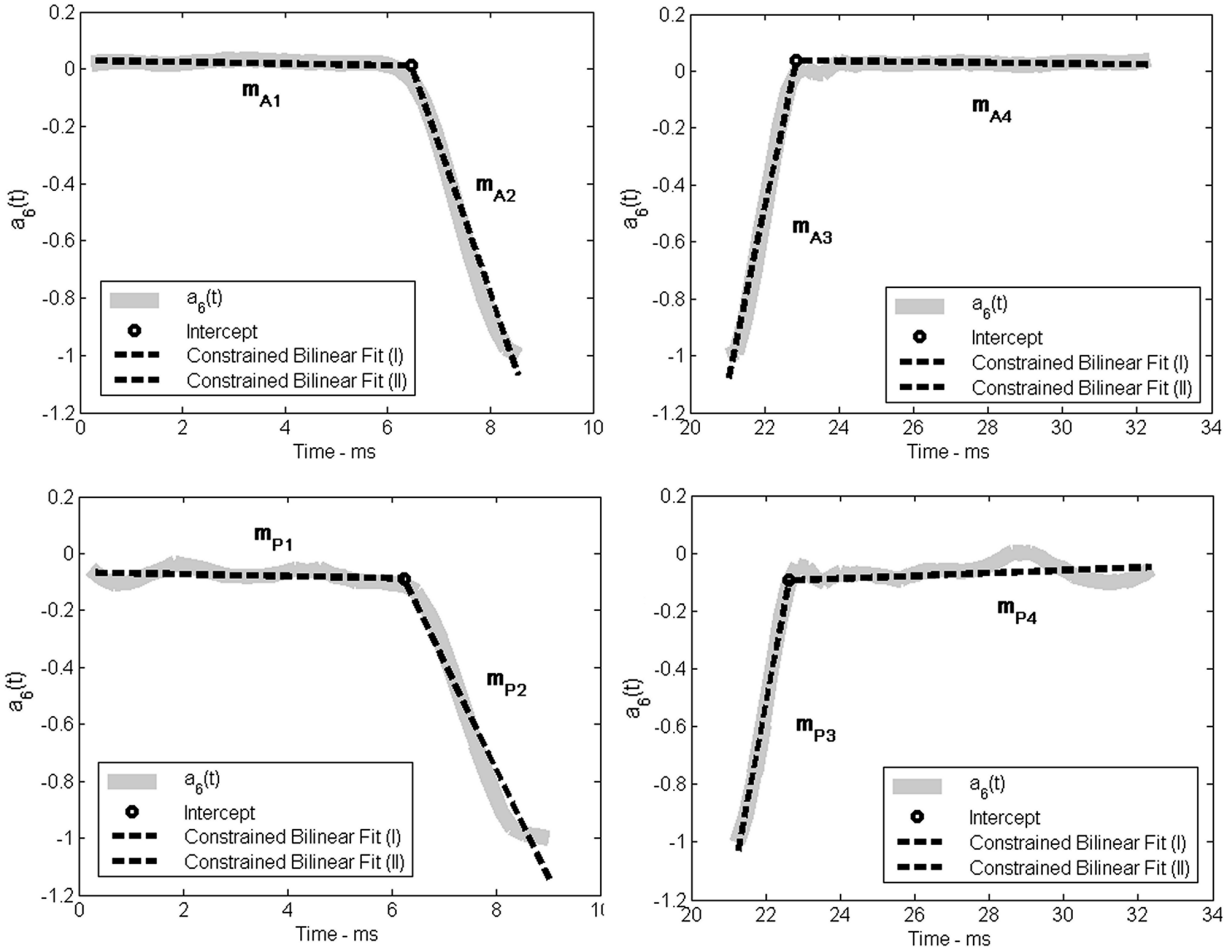
An example of bilinear fitting for the measurement of an individual from the young group of subjects. Upper row shows the first and the third stage of the corneal deformation, expressed in terms of the highest Chebyshev polynomial coefficient *a*_6_(*t*), for the anterior surface while the bottom row shows the corresponding stages for the posterior corneal surface. Dashed lines indicate the bilinear fit to each of the stages.

To separate the slow and fast changes in corneal deformation, each of the considered two stages has been quantitatively described by a constrained bilinear fit. This results in further division of each of the stages into two separate phases. Unlike in a piece-wise linear regression, in the constrained bilinear fit the estimate of the second line is conditioned on the estimate of the first line resulting in a model without discontinuities [31]. An example of the constrained bilinear fitting is shown in Fig 4.

The performance of bilinear estimation procedure has been investigated using nonparametric bootstrap in which residuals between the considered phase time series and the bi-linear model were resampled. The bootstrap estimated standard deviation of the intercept was small and amounted to less than a sample point which is equivalent to less than 0.24 ms. This indicates that the proposed procedure of bilinear fitting is justified.

As a result, we obtain eight slope coefficients *m*_*A*1_, *m*_*A*2_, *m*_*A*3_, *m*_*A*4_, *m*_*P*1_, *m*_*P*2_, *m*_*P*3_, *m*_*P*4_, where subscripts *A* and *P* denote the anterior and posterior surface, respectively and the numeral indicates the line order. The first line (*m*_*A*1_ or *m*_*P*1_) describes the initial dynamic state of the cornea surface (anterior or posterior, respectively) before the first applanation. The second and third lines (*m*_*A*2_ or *m*_*P*2_ and *m*_*A*3_ or *m*_*P*3_) indicate the ingoing and outgoing corneal dynamics, respectively. The last line (*m*_*A*4_ or *m*_*P*4_) describes the dynamics of the return to the original state of cornea. These parameters were considered for further statistical analysis. The same procedure was applied to both groups of subjects.

Since normality of the data was rejected (Jarque-Bera test, *P*<0.05), all slope coefficients were tested in relation to subject age with Wilcoxon rank sum test for equal medians. Additionally, of interest were relationships between the four sets of parameters (i.e., *m*_*A*1_ vs. *m*_*P*1_, *m*_*A*2_ vs. *m*_*P*2_, *m*_*A*3_ vs. *m*_*P*3_, and *m*_*A*4_ vs. *m*_*P*4_). These data was tested with Jarque-Bera normality test and the Bartlett's test for equal variances. Since in majority of cases normality and equal variance criteria were not fulfilled, non-parametric two-way ANOVA (Friedman) test was applied to examine the data for age dependence. The significance level for all tests was set at *α* = 0.05. All statistical analyses were performed in MATLAB.

## Results

Baseline characterization of both groups is shown in Table 1 (age, gender, CCT, and IOP). The group average IOP and CCT were (mean ± S.D.) 15.2 ± 1.9 mmHg and 559.4 ± 35.1 μm, respectively for the young group, and 14.8 ± 2.3 mmHg and 554.9 ± 36.8 μm, respectively for the older group. No statistically significant differences between age groups in CCT and IOP were observed (*p* = 0.39 and *p* = 0.66, respectively).

**Table 1:** Baseline group characteristics: age, gender, central corneal thickness (CCT) and intraocular pressure (IOP) in young and older subjects.

	Young Group	Old Group	*p*-value
**Age** (mean ± SD)	23 ± 3	58 ± 5	0
**Gender** (F/M)	11/14	11/14	NA
**CCT** [μm] (mean ± SD)	559.4 ± 35.1	554.9 ± 36.8	0.39
**IOP** [mmHg] (mean ± SD), [range]	(15.2 ± 1.9), [11.5, 19]	(14.8 ± 2.3), [10.5, 19]	0.66

Considering age-related changes in the slope coefficients (see Table 2), statistically significant differences were observed only for the *m*_*P*3_ parameter (Wilcoxon rank sum test, *p* = 0.009, *p* = 0.007, and *p* = 0.012 for *a*_6_(*t*), *a*_4_(*t*), *a*_2_(*t*), respectively). Table 3 presents a summary of statistical test results for two-way comparison, in which the corneal profile was used as a dependent variable. Statistically significant differences (two-way ANOVA (Friedman)) were observed for *m*_*A*3_ vs. *m*_*P*3_ (*p* = 0.016). Table 3 includes also the results of differential analysis where statistically significant changes were found for *dm*_3_ = *m*_*A*3_−*m*_*P*3_ (*p* = 0.017 and *p* = 0.005 for *a*_6_(*t*) and *a*_4_(*t*), respectively).

**Table 2:** Results (*p*-values) for testing age-related differences in slope parameters of the bilinear models. Subscripts *A* and *P* correspond to the anterior and posterior corneal profiles, respectively.

Wilcoxon rank sum test				
	*Slope*	*a*_6_(*t*)	*a*_4_(*t*)	*a*_2_(*t*)
**Anterior**	*m*_*A*1_	0.628	0.509	0.698
	*m*_*A*2_	0.426	0.831	0.742
	*m*_*A*3_	0.938	0.614	0.393
	*m*_*A*4_	0.313	0.244	0.116
**Posterior**	*m*_*P*1_	0.614	0.449	0.574
	*m*_*P*2_	0.174	0.207	0.684
	*m*_*P*3_	**0.009**	**0.007**	**0.012**
	*m*_*P*4_	0.907	0.561	0.892

**Table 3:** Results (*p*-values) of two-way ANOVA (Friedman) for testing age-related differences in slope parameters of the bilinear models. Subscripts *A* and *P* correspond to the anterior and posterior corneal profiles, respectively.

**Non-parametric 2-way ANOVA (Friedman) test**			
*Slope*	*a*_6_(*t*)	*a*_4_(*t*)	*a*_2_(*t*)
*m*_*A*1_ vs. *m*_*P*1_	0.476	0.945	0.902
*m*_*A*2_ vs. *m*_*P*2_	0.124	0.459	0.593
*m*_*A*3_ vs. *m*_*P*3_	0.055	0.121	**0.016**
*m*_*A*4_ vs. *m*_*P*4_	0.528	0.681	0.222
**Wilcoxon rank sum test**			
*Slope*	*a*_6_(*t*)	*a*_4_(*t*)	*a*_2_(*t*)
*dm*_1_ = *m*_*A*1_−*m*_*P*1_	0.858	0.749	0.793
*dm*_2_ = *m*_*A*2_−*m*_*P*2_	0.434	0.332	0.698
*dm*_3_ = *m*_*A*3_−*m*_*P*3_	**0.017**	**0.005**	0.051
*dm*_4_ = *m*_*A*4_−*m*_*P*4_	0.504	0.642	0.954

## Discussion

High-speed Scheimpflug images of the left eyes of 50 healthy volunteers in two age groups were used in the study. All subjects exhibited normal IOP values [32] and normal central corneal thickness values [33], which were not statistically different between the groups. The dynamics of the corneal deformation response was assessed in relation to subject’s age. We proposed the time-varying Chebyshev polynomial based model, in which higher even order terms exhibited a distinct three-stage dynamic behavior. Those even terms mostly capture corneal asphericity changes undergoing during cornea applanation. Likewise, the odd terms of the polynomial fit mostly capture the rotational movement of the eye globe. Focus was made on the first and the third stage of corneal deformation dynamics which include the times of the first and second corneal applanation. Those two stages were further optimally sequenced (by fitting a constrained bilinear model) into phases that were linearly modelled. The first phase represents the pre-applanation cornea state in which the applied air pressure is sufficiently small to cause only linear deformation of cornea. The second phase includes the first applanation and the peak air pressure value. The third phase corresponds to the second applanation of the cornea while the fourth phase is related to the return of corneal surface to its original state where again linear approximation to cornea deformation can be used.

Considering each surface separately, parameters of bilinear model showed age-related differences, particularly in the third phase of the posterior corneal profile where those differences were statistically significant. This confirms the preliminary results reported in [28]. On the other hand, when the anterior and posterior surfaces were considered as dependent factors in the analysis, statistically significant age-related changes were evident in the second and third phases for the 5% trimming of the profile data but not for one corresponding to the 10% trim. This suggests that the 5% trim may not be sufficient or that changes in corneal asphericity may play some role in this result. On the other hand, differential analysis, which carries more sensitive information, indicates statistically significant differences in the third phase for the 10% trim but not for the 5% trim thus confirming the results of previous analysis.

It is worth noting that in the first and the fourth phase of the corneal deformation dynamics the applied outer pressure is relatively low to cause any substantial deformation of the corneal profile and to show any age-related differences. In the second phase the outer pressure applied to the cornea is substantially higher than that of the IOP and it includes the first applanation. Our results indicate that the corneal deformation dynamics in that phase do not change with age. We conclude that the pressure applied to the cornea is sufficiently high for the corneal biomechanical properties to play a substantial role in that dynamics. Finally, age-related changes in biomechanical parameters of the cornea are evident in the third phase of corneal deformation dynamics where the outer pressure earlier applied to the cornea is being released.

It is now well established that the material properties of cornea depend on age [8–14]. Elsheikh et al. [4] showed statistically significant differences in corneal stiffness in relation to age. Tonnu et al. [14] demonstrated that subject age has a differential effect on the IOP measurements made by the Goldmann applanation tonometer (GAT) and ocular blood flow tonograph (OBF) compared to the handheld tonometer. Klein et al. [34] suggested that age could be associated with an overestimation of IOP value. Kotecha et al. [8] found the effect of age on IOP measurement suggesting an age-related corneal biomechanical change that may induce measurement error additional to that of CCT. The observed in our study age-related changes in corneal deformation confirm those earlier ex- and in-vivo studies and provide a new insight into particular phases of corneal deformation dynamics.

Various age-related changes in the biomechanics of ocular components have been reported in the literature [35–39]. Full knowledge of the aging processes in the cornea could bring an important insight into diagnosis and treatment of eye diseases. Numerical analyses of the corneal deformation dynamics incorporating subject’s age can be used to build a biomechanical model of the cornea that subsequently could help those endeavours.

## Supporting Information

S1 Raw Data An Excel file containing the bi-linear fitting slope parameters values for all even order time-varying Chebyshev polynomial coefficients analysis (*a*_6_(*t*), *a*_4_(*t*) and *a*_2_(*t*)). (XLSX)
